# Large perturbations in CO_2_ flux and subsequent chemosynthesis are induced in agricultural soil by the addition of elemental sulfur

**DOI:** 10.1038/s41598-017-04934-9

**Published:** 2017-07-05

**Authors:** Brian P. Kelleher, Paul V. Flanagan, Kris M. Hart, Andre J. Simpson, Seth F. Oppenheimer, Brian T. Murphy, Shane S. O’Reilly, Sean F. Jordan, Anthony Grey, Aliyu Ibrahim, Christopher C. R. Allen

**Affiliations:** 1 School of Chemical Sciences, Dublin City University, Glasnevin, Dublin 9 Ireland; 2 The School of Biological Sciences, Queen’s University Belfast, Medical Biology Centre, Lisburn Road, Belfast, BT9 5AG Northern Ireland; 3 Department of Chemistry, Division of Physical and Environmental Science, University of Toronto at Scarborough, 1265 Military, Trail, Toronto, Ontario M1C 1A4 Canada; 4 Department of Mathematics and Statistics, Shackouls Honors College, Mississippi State University, Mississippi State, Mississippi 39762 USA

## Abstract

The microbial contribution to soil organic matter has been shown to be much larger than previously thought and thus it plays a major role in carbon cycling. Among soil microorganisms, chemoautotrophs can fix CO_2_ without sunlight and can glean energy through the oxidation of reduced elements such as sulfur. Here we show that the addition of sulfur to soil results in an initial surge in production of CO_2_ through microbial respiration, followed by an order of magnitude increase in the capture of carbon from the atmosphere as elemental sulfur is oxidised to sulfate. *Thiobacillus spp*., take advantage of specific conditions to become the dominant chemoautotrophic group that consumes CO_2_. We discern the direct incorporation of atmospheric carbon into soil carbohydrate, protein and aliphatic compounds and differentiate these from existing biomass. These results suggest that chemoautotrophs can play a large role in carbon cycling and that this carbon is heavily influenced by land management practises.

## Introduction

Traditionally, humic substances were thought of as the main repository of organic carbon in agricultural soils. However, studies show that most soil biomass is actually present as bacterial and fungal cellular material^1, 2^. This is a critical observation: microbial biomass, unlike humic materials, can potentially play a direct role in global carbon cycles, actively releasing or trapping greenhouse gases, such as CO_2_ and CH_4_, under specific conditions. As one third of greenhouse gas emissions are associated with agriculture and soil accounts for more than half of this carbon^3^ there is a need to understand the influence of agriculture on carbon fluxes modulated by soil microbes^4^.

The contribution of photoautotrophic microorganisms to CO_2_ uptake, is well known^5^ and there is now a realisation that they play a significant role in the sequestration of soil carbon^6^. However, the contribution of chemoautotrophic microorganisms, through chemosynthesis or “dark carbon fixation” (DCF) has been generally neglected as a pathway to carbon assimilation^7^. Despite this, recent studies show that DCF is a quantitatively important CO_2_ uptake mechanism^8–11^.

Chemoautotrophy describes metabolic processes that are used to convert CO_2_ into organic materials (biomass) by microbes, using energy obtained from the oxidation of reduced organic or inorganic molecules. Unlike photoautotrophs, energy from light is therefore not needed for CO_2_ fixation. Chemoautotrophs can be further subdivided into two groups: 1) The chemolithotrophs, that use inorganic electron donors (e.g. NH_4_^+^, NO^2−^, Fe^2+^ and S_2_O_3_^2−^) to provide energy while fixing CO_2_; 2) The mixotrophs – a group of organisms that are facultative chemoautotrophs, i.e. assimilating CO_2_ but also using heterotrophic carbon metabolism when the right environmental circumstances apply.

Previously, we showed that the addition of thiosulfate to soils resulted in an order of magnitude increase in the uptake of CO_2_ by chemoautotrophs^12, 13^. Thiosulfate acts as an electron donor and provides the energy for a large increase in CO_2_ uptake. The uptake events occurred over the space of 48 hours in conditions conducive to chemoautotrophic growth.

Following on from these experiments, we now apply elemental sulfur (S°), rather than thiosulfate to an agricultural soil by incubating both an unaltered (SU) and an S° amended arable soil (SSA) for 12 weeks. The application of S° to agricultural lands is widespread as a nutritional supplement^14–16^. The incubations were carried out in both ^12^CO_2_ and ^13^CO_2_ atmospheres under close to natural CO_2_ concentrations (~400 ppm). The efflux (CO_2_ production through respiration and degradation), CO_2_ uptake and net change in atmospheric CO_2_ caused by the incubated soils was continuously measured. Critically, the use of stable isotopes in these experiments augments all aspects of the analysis; the tracking of CO_2_ from the atmosphere into soil biomass through a combination of DNA-Stable Isotope Probing (SIP), Nuclear Magnetic Resonance (NMR) and phospholipid fatty acid (PLFA) analysis to provide complimentary evidence of the effect of sulfur fertilisation.

## Results

### CO_2_ efflux and soil pH and nutrient level variations

**Figure 1:**
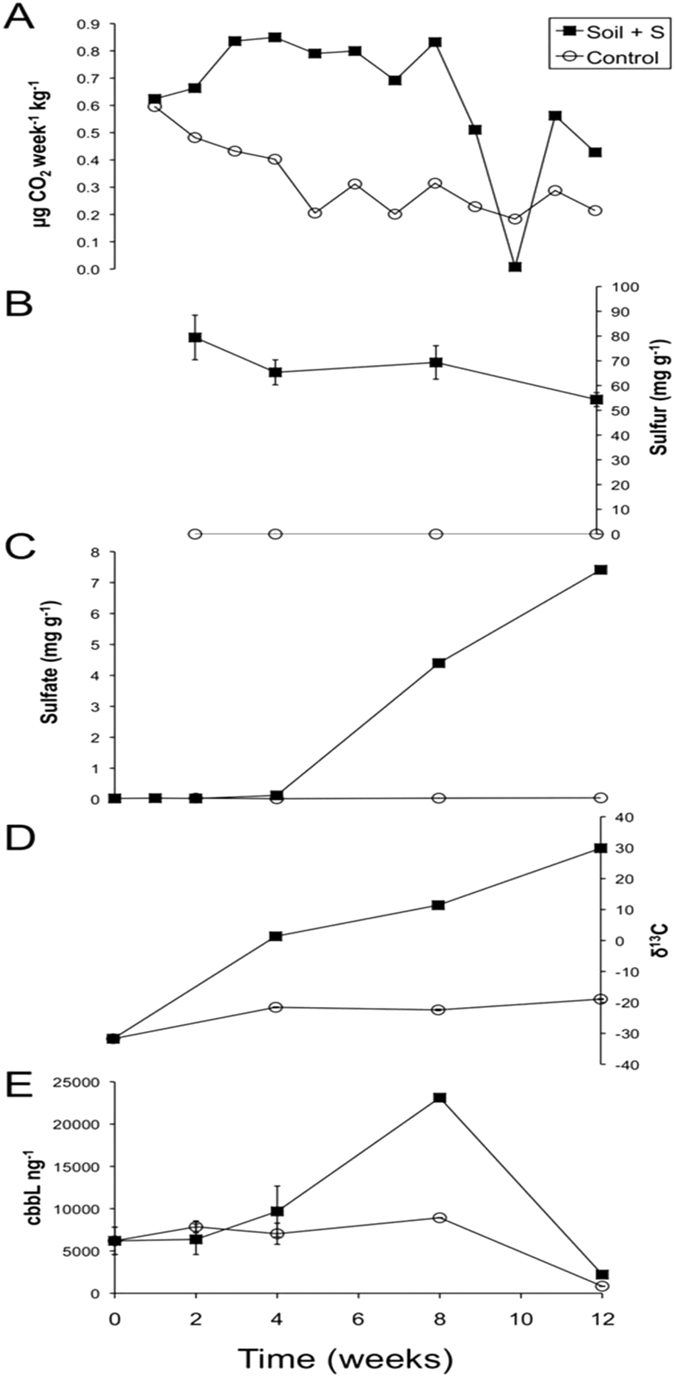
Variation in soil parameters between sulfur amended (S^SA^) and untouched soil (S^U^) over 12 weeks. (**A**) Average efflux rates of CO_2_ from both soils over time (Methods, Supplementary Tables 1 and 2 and Supplementary Information section 1). Positive values indicate CO_2_ efflux from the soil surface into the chamber atmosphere. (**B**) Sulfur concentration in both soils, (**C**) sulfate concentration in both soils, (**D**) average δ^13^C values (‰) of microbial PLFAs (average StDev ≤ 1.00‰) in both soils (Supplementary Information Table 3) and (**E**) RubisCO gene copy (*cbbL*) concentrations for both soils.

Figure 1 outlines the trends of some of the soil characteristics that were monitored over the 12 weeks. All experiments provide independent evidence of a stimulated soil microbial community caused by S° addition, especially from week 8 onwards. In general terms, our data indicates that sulfur addition at first results in a doubling of CO_2_ efflux (Fig. 1A, Supplementary Fig. S1, Supplementary Tables S2 and S3, Supplementary Discussion section 1). This pattern of higher CO_2_ efflux remained relatively consistent until weeks 8–9 indicating an increased rate of metabolic activity due to the stimulatory effect of added S°. The average CO_2_ efflux rate then rapidly decreases after week nine, and if we isolate this period (weeks 9 to 11) there was a net uptake of CO_2_ which coincided with the oxidation of sulfur (Fig. 1B) to sulfate (Fig. 1C). Both elemental sulfur and sulfate were below the limits of detection in the S^U^ soil (Fig. 1B and C). Uptake of CO_2_ is also reflected via isotopic enrichment of PLFAs showing a much higher incorporation of ^13^CO_2_ into the S° amended soil (Fig. 1D, Supplementary Discussion section 2 and Supplementary Table S4). Additionally, Fig. 1E shows that the RubisCO gene (*cbbL*), (Supplementary Discussion section 3) associated with atmospheric CO_2_ uptake through the RubisCO pathway^17, 18^, increased by an gene copy number order of magnitude at week 8 in the S^SA^ soil, coinciding with the CO_2_ uptake event (Fig. 1A) and S° oxidation (Fig. 1B and C).

CO_2_ efflux rates increased again by week 11 but did not return to the relatively high levels observed between weeks 2–9. The CO_2_ efflux data for weeks 11–12 suggests a steady state has been achieved after the rapid expansion of the chemoautotrophic population after week 8. Although the CO_2_ data indicates a reduced capacity for CO_2_ efflux after the point event of week 10, comparison with the control soil (S^U^) still suggests a stimulated autotrophic microbial community mineralising/respiring carbon overall. Over the course of the incubations the pH profile of S^SA^ shifted by 1.8 units from a pH of 7.3 to 5.5 whilst there was no pH change in S^U^ (Supplementary Fig. S2). The drop in pH is likely due to the oxidation of S° to SO_4_^2−^ ^19^ carried out by a range of microorganisms^20^. Associated with the drop in pH is an increase in available soil micronutrients particularly, Zn, Cu, Fe and Mn reflecting higher solubility at lower pH^21^.

### Microbial Community Changes

**Figure 2:**
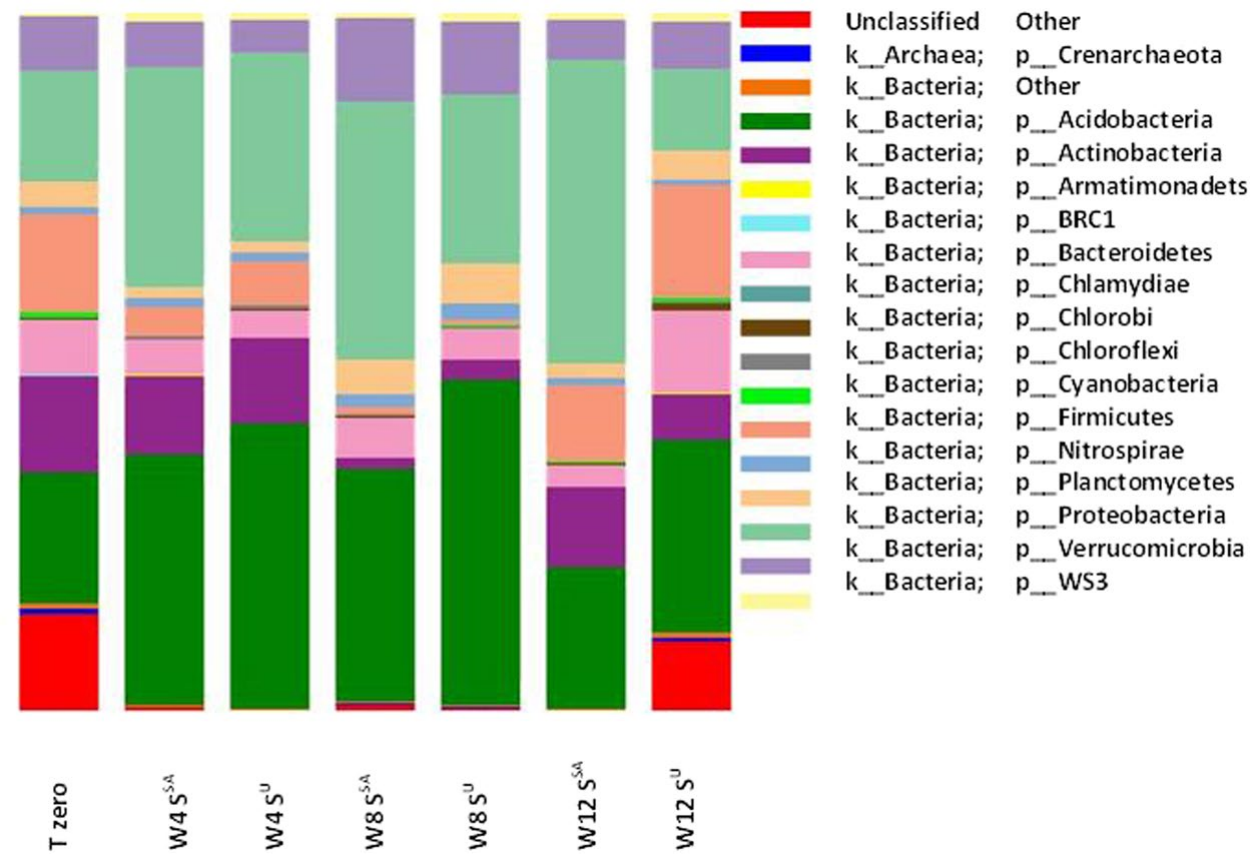
Microbial phylum abundance determined through pyrosequence analysis of 16S rRNA genes at time zero and following 12 weeks of incubation under both sulfur amended (S^SA^) and untouched soil (S^U^). Phyla were determined by analysis through the Quantitative Insights Into Microbial Ecology (QIIME) associated pipelines^56^. Taxonomy was then assigned using the GreenGenes reference database^66^. T = time, W = week, S^SA^ = Sulfur amended soil and S^U^ = unaltered soil.

Most work on microbial chemoautotrophy has focussed on the use of classical microbiological approaches to isolate and study microbes from environmental sources in the laboratory^22–24^. Microbiologists have deciphered the genetic and biochemical components and mechanisms that chemoautotrophs employ to fix CO_2_ through the culturable sub-set bacteria. The major divisions of chemoautotrophy were established through this work – significantly, the ability of chemoautotophs to utilise inorganic electron donors such as sulfur compounds was established in the 1980’s^25^. By employing metagenomic techniques here, we wanted to show how the total with microbial community changes during the addition of sulfur, and with variation in the composition and functioning of the microbial community over the 12 week experimental time frames. Prior to incubation, barcode pyrosequencing revealed that the most abundant, classified, phyla found in the soil used were *Acidobacteria*, *Proteobacteria*, *Firmicutes*, *Actinobacteria, Bacteroidetes*, *Verrucromicrobia*, *Planctomycetes* and *Nitrospirae* (as shown in Fig. 2, sample id TT0). However, the microbial population of S^SA^ and S^U^ changes over the course of the 12 week incubation (Fig. 2). In both soils there were large increases in the number of sequences representing the microbial phyla *Acidobacteria* and *Proteobacteria*. The *Proteobacteria* were consistently higher in S^SA^ peaking at week 12. Of this phyla almost half were identified at the genus level as *Thiobacillus spp*. Sequences attributed to *Thiobacillus spp* for S^U^ peak at only 0.1% of total microbial sequences. Their increased presence suggests that they thrived by oxidising the S° to SO_4_^2−^ and using the conserved energy to chemosynthetically capture carbon (Supplementary Discussion Section 4).

### DNA labelling provides insights into carbon fixing populations

To test the hypothesis that microbial community changes driven by S^0^ oxidation led to increased inorganic carbon capture, Stable Isotope Probing (SIP) was carried out by extracting DNA from both soils (under ^13^CO_2_ enrichment) over the course of the experiment and subjecting the samples to isopycnic ultra-centrifugation. SIP is a specific metagenomic technique that utilises carbon sources, such as ^13^CO_2_, that are labelled to differentiate between those components of a community that can use the substrate and those that cannot^26^. Nucleic acids are labelled in microorganisms that metabolise the compound, and this biological material can then be separated from unlabelled nucleic acids from other environmental microorganisms for study. The use of SIP methods to study free-living chemoautotrophy in soils has received relatively little attention^13, 27^.

DNA from both soils were subjected to isopycnic CsCl gradient ultracentrifugation (DNA-SIP) to separate the ‘heavier’ ^13^C-DNA with buoyant density of 1.71–1.73 g/ml, from the larger community ^12^C-DNA with a buoyant density of 1.66–1.68 g/ml. If the added sulfur is oxidised and facilitates the chemosynthetic uptake of carbon (^13^CO_2_) then there should be a ^13^C-labeled fraction of extractable DNA that can provide an insight into the identity of the chemoautotrophs. This ^13^C labelled fraction is apparent in the DNA isolated from the sulfur amended soil (S^SA^) at week 12 and recovered DNA from both incubations showed clear differences when probed through 16S rDNA directed qPCR. Nucleic acids isolated from the sulfur amended soil (S^SA^) at week 12 were enriched with ^13^C, as demonstrated through a shift in 16S rDNA gene profile after analysis of SIP fractions using RT-PCR (Supplementary Fig. 3). Unlabelled, control DNA (from T0) had maximum 16S rDNA gene copies in the 1.70 g.ml^−1^ fraction whereas maximum 16S rDNA gene copies were detected in the 1.725–1.74 g.ml^−1^ density fractions from nucleic acids extracted from S^SA^ at week 12. The microbial communities identified in the isotopically enriched fraction of the S^SA^ soil varied from the overall microbial population with the presence of only 4 bacterial classes; 62.7% *β Proteobacteria*, 15.3% *Actinobacteria*, 11.8% *Bacillus* and 10.2% *Thermoleophilia*. The most abundant genus in heavy fractions was *Thiobacillus spp* accounting for 100% of identified sequences affiliated with *β Proteobacteria* (Supplementary Discussion Section 5). Each of the bacterial groups in the isotopically enriched SIP fractions have been identified at class level as being potentially CO_2_ fixing bacteria as described by Saini *et al*.^28^. All DNA fractions generated through SIP experiments were also probed through PCR with primers specifically targeting the archaeal 16S rRNA gene^29^. At no point in the experiment were PCR products generated in “heavy” fractions of S^SA^. Archaeal products were detected in the total metagenomic DNA and “light” fractions of both soils throughout the experiment, but not in the labelled fractions, suggesting that, in this study, they did not participate in the chemosynthetic uptake of CO_2_. Interestingly, we also find that 16:1ω5, a signature PLFA biomarker for arbuscular mycorrhizal fungi (AMF), is significantly enriched in ^13^C^30^. This may be due to their role as decomposers, predators and cross feeders but the possibility of direct uptake of CO_2_ cannot be ruled out.

Importantly, 16S rDNA gene abundances exhibited no shift in buoyant density from nucleic acids isolated from soil S^U^ following the same incubation period suggesting that no significant CO_2_ uptake had occurred. NMR and PLFA analysis show that CO_2_ was autotrophically fixed at very low concentrations by the microbial community in the unamended soil (S^U^). What is clear, however, is that the addition of sulfur has considerably enhanced chemoautotrophy in the soil.

### Tracking the fate of CO_2_ with Nuclear Magnetic Resonance (NMR)

**Figure 3:**
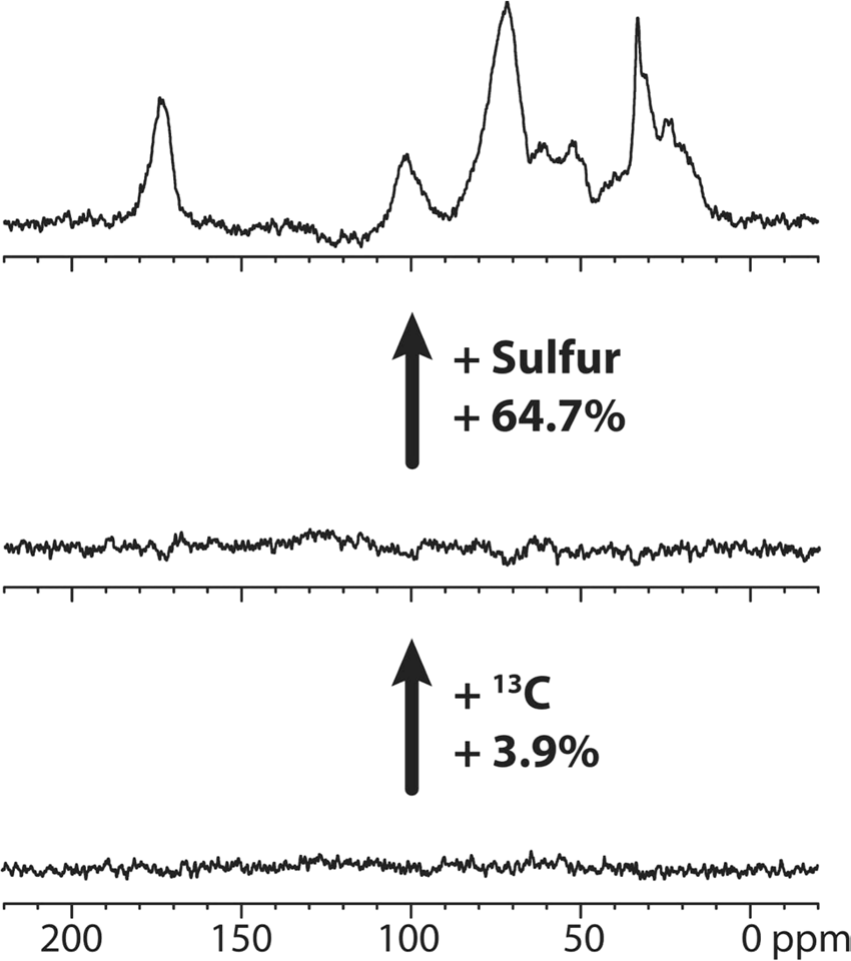
^13^C CP-MAS NMR difference spectra, showing the total carbon increase with ^13^C labelling. A (control with no ^13^C enrichment) to B (with ^13^C enrichment), 3.9% and B to C (with ^13^C enrichment and sulfur addition), 64.7%.

To assess the fate of captured carbon we subtracted ^13^C NMR spectra generated from organic extracts from both soils (S^U^ and S^SA^ **)** at week 12 to both quantify and identify carbon assimilated from the ^13^C enriched atmosphere^13^. The NMR data can be used to identify how the label is incorporated into the different chemical categories in the SOM. A quantitative comparison of the CP-MAS ^13^C spectra of the unaltered soil, S^U^ under ^12^CO_2_ and ^13^CO_2_ atmospheres shows that the total carbon signal in the soil increased by 3.9% during the 12 weeks (Fig. 3). This indicates that extant chemoautotrophs carry out chemosynthesis and under favourable conditions will slowly capture and integrate carbon into soil. When the natural abundance of ^13^C and isotopic enrichment of the labeling gas are considered this relates to approx. 0.04% (or ~1 in every 2500 carbons) of the total soil carbon coming from chemosynthesis in the 12 week period. After the addition of sulfur (S^SA^) the total carbon signal increases by approx. 64.7% corresponding to ~0.7% (or ~1 in every 150 carbons) of the total soil carbon coming directly from the chemosynthetic uptake of CO_2_. In simple terms, there was a 20 fold increase of carbon in the soil as a direct result of the addition of sulfur.

**Figure 4:**
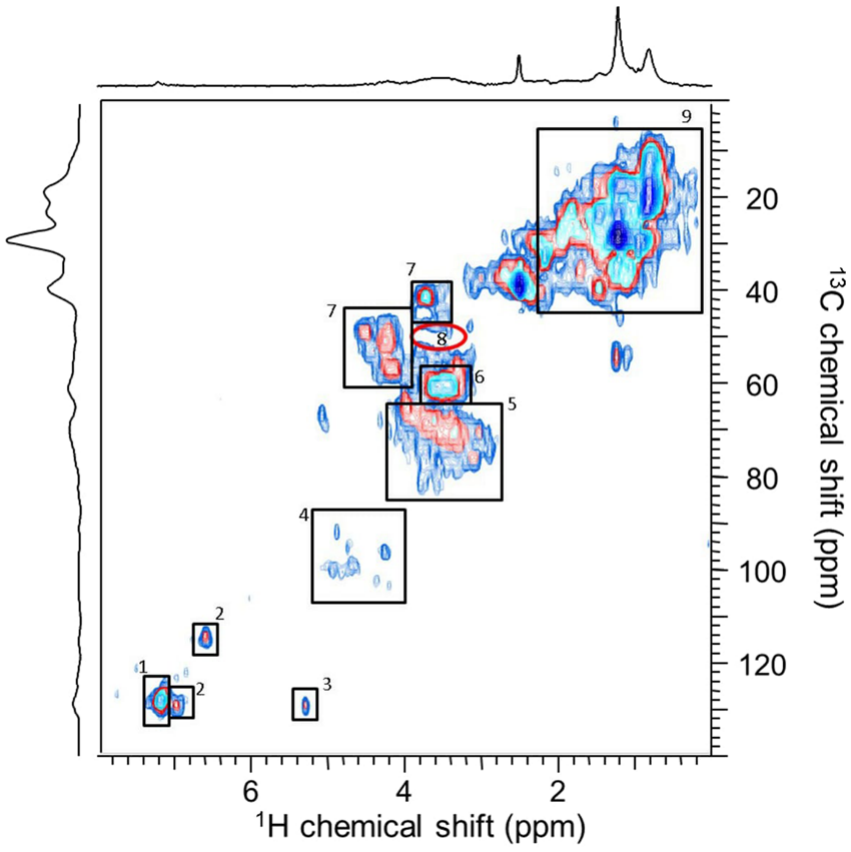
^1^H-^13^C HSQC difference spectrum showing only the components resulting from addition of sulfur in the DMSO swellable fraction. Assignments in 1, phenylalanine (from peptide/protein); 2, tyrosine (from peptide/protein); 3, unsaturations (HC=CH) in lipids, 4, anomeric protons (carbohydrates); 5, other CH in carbohydrates; 6, CH_2_ in carbohydrates; 7, α-protons in peptides and proteins; 8, methoxyl in lignin; 9, aliphatic linkages including signals from various lipids, and side chain protons in peptides.

Solid-state NMR provides an overview of the major types of bulk chemical functional groups, but the extraction of structural information is very challenging and in some cases impossible. However, high resolution magic angle spinning (HR-MAS NMR) can be employed to overcome challenges associated with pure solid-state NMR analyses. A solvent is added to the analyte, and, after swelling, the components become NMR observable. HR-MAS NMR allows the analysis of materials that swell, become partially soluble, or form true solutions to be analysed at resolution close to that observed in solution-state NMR^1, 31–33^. An HSQC spectrum is an experiment that detects H–C bonds within a structure^34^. A cross-peak represents the chemical shift of both carbon and proton atoms in a C–H unit. When considered together, the cross-peaks form a specific pattern or “molecular fingerprint” of a specific structure or class of structures. Figure 4 shows a ^1^H–^13^C Heteronuclear Single Quantum Coherence (HSQC) HR-MAS NMR difference spectrum between the sulfur amended and unaltered soils (S^U^ S^SA^ **)** at week 12^34, 35^. The only signals present in the difference spectrum are from chemical categories that increased due to the addition of sulfur. The spectrum is dominated by lipids, carbohydrates and protein/peptides arising from microbial biomass.

Comparison of relative integrals before and after and when the isotopic enrichment of the label is accounted for corresponded to ~0.88% of the lipids, 1.6% of the carbohydrates and 1.33% of the protein are labeled over the 12 week period in the HR-MAS observable swellable fraction. As expected, the lignin component (as represented by a methoxy carbon crosspeak at ∼3.7–55 ppm), often the most intense signal in SOM, is not present, considering this is exclusively synthesised as a structural biopolymer in plants^36^. The NMR results indicate that, provided with an electron donor, in this case sulfate, soil microbes are able to directly utilise the atmospheric CO_2_ source to produce a lipid component for metabolism, protein/peptide for growth, and carbohydrate likely being made for both purposes. The NMR spectra include contributions from amide, peptides, and dominant CH_3_ signals in the aliphatic region (Figs 3 and 4). These signatures are similar to microbial SOM isolated from temperate grasslands and are common in soil microbial extracts and characteristic of microbial organic matter^2, 37, 38^. NMR analysis of the control soil (S^U^) showed negligible isotopic labeling after 12 weeks exposure to ^13^CO_2_, indicating that the soil harbours chemosynthetic microbes but without an electron donor, their contribution to carbon flux is minimal^27, 39, 40^.

## Discussion

In this study we uniquely combine; (a) NMR and mass spectrometry (MS) approaches (b) metagenomic microbial population analysis, including SIP, (c) mathematical models and (d) RubisCO gene monitoring in a single experiment to; (i) accurately quantify the uptake of carbon into biomass *and* determine its fate, (ii) qualitatively establish the nature of chemotrophic microbes through SIP and (iii) quantify the uptake of CO_2_. The key finding here is that we have shown unequivocally that the addition of inorganic sulfur to soil has a marked effect of CO_2_ entrapment in the microbial biomass.

This study validates previous investigations where the application of sulfate to four soils also results in an order of magnitude increase in CO_2_ uptake^12, 13^. Conditions in those experiments were optimised for chemoautotrophic growth and as the sulfur applied was already oxidised the uptake event occurred within 48 hrs. In the present study sulfur was applied in elemental form to a fresh arable soil to reflect more natural conditions and agricultural practice. The uptake event again coincides with the availability of sulfate but is now preceded by a large increase of respired CO_2_, produced by microbes stimulated by the presence of elemental sulfur. Additionally, in our earlier work, the majority of the carbon was taken up and stored in the form of lipids as an energy store. In this present study which takes place over a period 12 weeks, it is clear the sequestered carbon is transferred to other microbial components (carbohydrates and proteins) and indeed the microbes are able to grow on carbon sequestered from the atmosphere. One of the aims of this work is to show that an inter-disciplinary approach can effectively be used to study the contribution of mixotrophs to CO_2_ capture in a typical agricultural soil. The paper cannot make any statements about the role of mixotrophs in all soils or even a particular subset of soils. Neither can the paper claim to be able to say that the methods are quantitatively comparable to other methods for soil CO_2_ uptake measurements. However, the study does represent a ‘step change’ in our ability to assess this fundamental process that has so many implications to a broad range of interests – from agriculturalists to those involved in green policy and trying to evaluate the potential for soils in carbon capture.

We have also demonstrated for the first time the importance of mixotrophic autotrophy over chemolithotrophy in the nutritional improvement of S^0^ augmented agricultural soil. Mixotrophs are understandably difficult to study *in situ*, as distinguishing the metabolism of these bacteria leading to CO_2_ fixation from heterotrophic catabolism is challenging. However, in this study we have demonstrated an approach that can be used to do this, and highlighted the importance of mixotrophs therefore in agricultural soils. One aspect of the research that does need further investigation is the nature of microbial transformation of S° to SO_4_^−2^. While S° is also oxidized by *Thiobacillus spp*. to SO_4_^−2^, it would be wrong to assume that in our case the Thiobacilli present were orchestrating both steps in the sulfur cycle. While autotrophic fixation of CO_2_ is likely to be linked to the sulfate reduction, the oxidation of inorganic sulfur may not yield enough energy to facilitate CO_2_ fixation directly, and is probably linked to heterotrophic metabolism even in *Thiobacillus spp*. – which would not be picked up through the SIP experimentation^19^.

It is now recognised that autotrophic CO_2_ fixation is one of the most important processes in the carbon cycle. However, despite the ubiquitous nature of chemoautotrophs we know virtually nothing about the CO_2_ entrapment capabilities of these microorganisms. We know very little about the role these organisms play in commercially and environmentally significant locations – such as agricultural soil. However, here we show that chemosynthetic microorganisms can be quantitatively important in the uptake and release of CO_2_ and that our management of soil plays a large role in carbon cycling. Given that about 50% of all the Earths biomass is present in soils and sediments as bacteria, it is certain that much of this biomass contains CO_2_ fixing potential^8, 41^. This potential needs to be understood and exploited in any effective global carbon management strategy.

## Methods

### Sampling

Surface soil (0–10 cm depth) was collected aseptically (approximately 5 kg) from an arable field used for crop growth on the outskirts of Dublin, Ireland (coordinates; 53.4332° N, 6.3229° W, sampled on 15^th^ Feb. 2012), and immediately transferred to the laboratory. The soil was classified as a Luvisol with a fine loamy texture (http://gis.teagasc.ie/soils/map.php). Soil properties include a pH of 7.23 ± 0.12, %C; 4.57, %H; 0.65, %N; 0.31 and %P; 0.14.

### Soil Incubations

The soil was divided into four sterile containers (620 g each) and two of the soil samples were amended with elemental sulfur (S^SA^). The un-amended soil samples (S^U^) were used as controls and to determine basal respiration. All soils were incubated in a custom built Environmental Carbon Dioxide Incubation Chambers (ECIC) for a period of 12 weeks^12, 13^. The S^U^ and S^SA^ soil samples were exposed to an atmosphere that contained ^13^CO_2_ at a concentration of 400 ppm (±~40 ppm) for the duration of the incubation. These samples were used for isotopic chemical and microbiological analysis (SIP, NMR and GC-MS-IRMS). The second S^U^ and S^SA^ soils were exposed to a ^12^CO_2_ atmosphere under exactly the same conditions. These experiments were used to track the quantity of CO_2_ in the atmosphere and to demonstrate the effect of adding sulfur on CO_2_ efflux. For detailed information on the operation and principles of the ECICs please see Hart *et al*., 2013a^12^. Incubations were carried out in a temperature of 10.4 °C, representing the average air temperature in Ireland based on Met Eireann data (http://www.met.ie/climate-ireland/surface-temperature.asp). Sub samples were taken, at weeks 0, 2, 4, 8 and 12 for molecular biology and chemical analysis. In each case 50 g of sample was removed and stored immediately at −20 °C prior to analysis. All experiments were conducted in the dark so as to negate interference of CO_2_ flux by photoautotrophs and plant growth.

The ECIC was vented every second day and levels of ^13^CO_2_ were replenished to original concentrations to avoid dilution of the labelled CO_2_ with unlabelled CO_2_ that may originate from soil respiration. During venting the soils were sprayed with water to keep the soil moist (approximately 12.5 ml per week). Prior to elemental sulfur amendment the sulfur was treated with UV light overnight in a sterile container in order to minimise microbial contamination. The sulfur (Sigma-Aldrich) was added at a concentration of 20 Kg.ha^−1^ in accordance with the Sulfur Institute guidelines, which suggest the addition of between 15–30 Kg.ha^−1^ of elemental sulfur for grass and cereal crop growth (http://www.sulfurinstitute.org/learnmore/faq.cfm#need).

### CO_2_ efflux calculations and statistics

For gas flux calculations and statistics please see Hart *et al*., 2013a; Hart *et al*., 2013b^12, 13^. Briefly, high resolution [CO_2_] data consisting of measurements taken every 30 sec were taken throughout each incubation. We treat the concentration of CO_2_ in the chamber in ppm as a function of time, where time was measured in minutes, C(t). We consider only two mechanisms that can cause a change in the CO_2_ concentration in the chamber, 1) outgassing, 2) some soil/organism action that is either taking up or releasing CO_2_.

We assumed outgassing occurred at a rate proportional to the difference in CO_2_ concentration in the chamber and in the external environment with a constant of proportionality of *h*_*c*_ with units of 1/minutes. We computed this constant five times using five different sets of experiments. This resulted in five estimates of *h*_*c*_ that were remarkably close, an indication that the model is robust. The *h*_*c*_ we use below in our model is obtained by using the data from all five of these independent experiments. When we include the soil/organism action our full model is:

1 $$\frac{dC}{dt}={h}_{c}(\bar{C}-C(t))+f(t)$$

Where ($\bar{C}$) is the external CO_2_ concentration (C) is the internal CO_2_ concentration, and (f) is an unknown source/sink of CO_2_. External CO_2_ concentration (the laboratory atmosphere) varies over time according to unpredictable environmental conditions. The laboratory atmospheric flux of CO_2_ (in the vicinity of the ECIC) was measured in three sequential experiments using a pSense portable CO_2_ detector (AQ Controls Ltd. Stomnevagen, Sweden), each lasting for 7 days to determine the mean concentration (data not shown). We treat the external CO_2_ concentration as constant using the average external CO_2_ flux (data not shown), fixing it at a mean value $\bar{C}$ = 401.6 ppm. Final conversion factor for ppm to gram (g) is given as ((xvd)/10 × 10^5^). Where, chamber volume (v) is 40.059 l, density (d) of 1.97 g l^−1^, and x is CO_2_ (ppm). Soil respiration was incorporated into the correction factor when determining uptake rates at a particular partial pressure. Positive values indicate net efflux of pCO_2_ over time and conversely, falling rates of efflux represent uptake. Rates are calculated as a direct computation, not an average of data points ((net change in CO_2_ at final time - net change in CO_2_ at initial time)/elapsed time). Using our original work, get a mean value for ${h}_{C}={\rm{3.7454}}\times {{\rm{10}}}^{-4}$. We now can calculate the unknown function *f* using the data at our disposal and our identified $\overline{C}$ and *h*_*C*_. There are a variety of ways we can do this, we will however use a centered difference approach to finding the derivative of *C*:

$$\begin{array}{rcl}\frac{dC}{dt}(t) & \approx  & \frac{C(t+0.5)-C(t-0.5)}{1}\\  & = & C(t+0.5)-C(t-0.5)\end{array}$$

At time 0 and the final time we use a forward and a backward difference,

$$\begin{array}{rcl}\frac{dC}{dt}(0) & \approx  & \frac{C(0.5)-C(0)}{0.5}\\  & = & 2(C(0.5)-C(0))\\ \frac{dC}{dt}({t}_{final}) & \approx  & \frac{C({t}_{final})-C({t}_{final}-0.5)}{0.5}\\  & = & 2((C{t}_{final})-C({t}_{final}-0.5))\end{array}$$

Thus we have, for the times other than time 0 and the final time:

$$C(t+0.5)-C(t-0.5)\approx {h}_{c}(\overline{C}-C(t))+f(t)$$

We turn this into an equality to solve for an approximate value of *f*, the rate of change due to soil/organism action and

$$f(t)=C(t+0.5)-C(t-0.5)-{h}_{c}(\overline{C}-C(t))$$

At the endpoints

$$\begin{array}{c}f(0)=2(C(0.5)-C(0))-{h}_{c}(\overline{C}-C(0))\\ f({t}_{final})=2(C({t}_{final})-C({t}_{final}-0.5))-{h}_{c}(\overline{C}-C({t}_{final}))\end{array}$$

Of interest is that the overall mass change due an unknown process is obtained by integrating *f* and multiplying by the conversion factor 7.1613e-005 to obtain the net mass change due to the unknown process. The integration is carried out using the trapezoid rule. For a more detailed account of the algorithm, please see Hart *et al*., 2013a. Treatment differences were compared using 1-way ANOVA and all graphs were produced using SigmaPlot version 11.0, from Systat Software, Inc., San Jose California USA.

### Nuclear Magnetic Resonance

Pre-treatment and analysis of soils by Solid State ^13^C NMR and High Resolution-Magic Angle Spinning (HR-MAS) NMR was carried out as in our previous publication, Hart *et al*., 2013b^13^.

#### Solid State ^13^C NMR Analysis

For solid state ^13^C analysis, 100 mg samples were packed into 4-mm zirconium oxide rotors with Kel-F rotor caps. ^13^C cross-polarization with magic angle spinning (CP-MAS) NMR spectra were acquired using a Bruker Avance III 500 MHz spectrometer (Bruker Biospin, Canada) equipped with a Bruker 4-mm H-X MAS probe. Spectra were acquired at 298 K with a spinning rate of 13 kHz, a ramp-CP contact time of 1 ms, 1 s recycle delay, 8192 scans, 1024 time domain points, and ^1^H decoupling using Spinal 64. Spectra were processed using the Bruker Topspin software (version 2.1) with a filling factor of 2 and exponential multiplication resulting in a line broadening of 30 Hz in the final transformed spectrum. Spectral subtractions to produce the difference spectra were performed in the interactive mode of Topspin 2.1.

#### High Resolution*−*Magic Angle Spinning (HR-MAS) NMR Analysis

Prior to NMR analysis, samples and materials that came into direct contact with the samples (zirconium oxide rotors, Kel-F caps, Kel-F sealing rings, steel spatula, and pipet tips) were dried for one week over phosphorus pentoxide (P_2_O_5_) under vacuum at room temperature to reduce traces of molecular water that would interfere with NMR spectra. A 40-mg dry sample was then weighed directly in a 4-mm zirconium oxide rotor and 60 μL of DMSO-d_6_ was added as a swelling solvent. After homogenization using a stainless steel mixing rod, the rotor was doubly sealed using a Kel-F sealing ring and a Kel-F rotor cap. HR-MAS NMR spectra were acquired using a Bruker Avance III 500 MHz spectrometer (Bruker Biospin) equipped with a Bruker 4-mm triple resonance (^1^H, ^13^C, ^15^N) HR-MAS probe with an actively shielded Z gradient and a spinning speed of 6.66 kHz. All HRMAS experiments were acquired at 298 K. Proton (^1^H) experiments were acquired with 256 scans, 4096 time domain points, and a recycle delay of 2 s. Solvent suppression was achieved by presaturation utilizing relaxation gradients and echoes^42^. ^1^H HR-MAS spectra were processed with a zero- filling factor of 2 and exponential multiplication, resulting in a line broadening of 2 Hz in the transformed spectrum. ^1^H-^13^C heteronuclear single quantum coherence (HSQC) spectra were collected in phase sensitive mode using Echo/Antiecho-TPPI gradient selection but without sensitivity enhancement. Scans (2048) were collected for each of the 96 increments in the F1 dimension. A relaxation delay of 1 s was employed with 1024 time domain points collected in F2 and a 1 J ^1^H-^13^C of 145 Hz. The F2 dimension was multiplied by an exponential function corresponding to a 15 Hz line broadening while the F1 dimension was processed using sine-squared functions with phase shifts of π/2. Both dimensions were zero-filled by a factor of 2. Spectral Integration from HSQC was done in the multi-integration mode of AMIX 3.8.7. Due to variations in ^1^H-^13^C couplings HSQC cannot provide absolute quantification, but when data are collected using identical acquisition and processing parameters, the approach can be used to monitor relative change between samples^43^. Regions were defined as follows: protein (phenylalanine resonance) ^1^H 7–7.3 ppm, ^13^C 125–130 ppm; lignin (methoxy signal) ^1^H 3.6–3.8 ppm, ^13^C 54–58 ppm; carbohydrates (CH_2_ signal) ^1^H 3.4–3.6 ppm, ^13^C 58–63 ppm; lipids (CH_2_ β to COOH), ^1^H 1.1–1.33 ppm, ^13^C 26–32 ppm. Multidimensional NMR of SOM including detailed assignments of the microbial fraction has been considered in detail in previous publications^1, 44–47^. Assignments are confirmed by NMR spectral predictions (Advanced Chemistry Development’s ACD/SpecManager and ACD/2-D NMR Predictor using neural network prediction algorithms).

NMR spectroscopy has been shown to be highly reproducible >99%^48^. Furthermore soils were extensively homogenized to make a representative average sample at each time. Considering the relatively large sample size 100 mg for solid-state NMR and 40 mg for HR-MAS NMR, the NMR data represent an average overview of the carbon functionalities throughout the sample.

### DNA extraction, PCR qPCR and DGGE

DNA was extracted from 0.3 g soil samples using a PowerSoil DNA extraction kit (Mobio) according to manufacturer guidelines. DNA was stored at −20 °C prior to analysis. All standard PCR reactions were carried out in 25 µL volumes using a DNA Engine DYAD Peltier Thermal Cycler (BioRad). In the case of the 16S rRNA gene directed PCR primer pair, P1/P2 were used targeting the V3 region of the bacterial 16S gene^49^. RubisCO genes associated with chemoautotrophic CO_2_ fixation were targeted using primer pair *cbbL*f/r^50^.

The abundance of 16S rRNA and *cbbL* genes was quantified by qPCR using primer pair P1/P2 or *cbbL*f/r respectively. The thermal cycling program consisted of an initial hotstart at 95 °C for 10 min, followed by 35 cycles of 95 °C for 20 s, 58 °C for 20 s and 72 °C for 20 s. Plate reads were taken following each extension step at 72 °C. Gel electrophoresis (Biorad) of products was performed to ensure specificity of product and in the construction of standards. Once product identity was confirmed melting curves were performed at the end of each qPCR run to confirm reaction specificity. All Quantitative PCR reactions were performed in an Opticon 3 real-time PCR machine (Biorad) using the Maxima SYBR Green I MasterMix (Fermentas). Each 25 µL reaction contained 1 µL of template DNA and a final primer concentration of 0.35 µM each.

Standard curves, for gene quantification, were generated using purified PCR products from *Pseudomonas putida* ^51^ or environmental amplicons for 16S rRNA and *cbbL* respectively. Briefly, PCR products from *P. Putida* (DSMZ Culture collection number 8368) or environmental samples were excised from an agarose gel, pooled together and purified using an agarose gel PCR purification kit (Fermentas). DNA concentrations were then measured spectrophotometrically at 260 nm using a microcell cuvette (Hellma). Copy numbers were calculated according to the size of the amplicon (Whelan *et al*.)^52^. The standard curves were linear over 5 orders of magnitude with an r^2^ value >0.95. Amplification efficiency was calculated as ranging between 91–97%. Results are expressed as copy number per microgram of DNA extracted from each soil sample.

### Isopycnic CsCl gradient ultracentrifugation

Isopycnic centrifugation was carried out according to Neufeld *et al*.^53^ following the gradient fractionation method. Gradients for Isopycnic ultacentrifugation were prepared by dissolving CsCl in ultrapure H_2_O to a final concentration of 1.89g.ml^−1^ according to refractive index (RI) measurements (Atago-R-5000). Gradients were then loaded with ~5 ng of metagenomic DNA. Ultracentrifugation (Beckman-Coulter Optima TLX) was performed at 395,833 g for 16 h at 20 °C^13^. Fractionation and precipitation of DNA extracts was carried out as described by Neufeld *et al*.^53^. Fractions were monitored for the presence of DNA through 16S qPCR using primers P1 (5′-CCTACGGGAGGCAGCAG-3′) and P2 (5′-ATTACCGCGGCTGCTGG-3′)^49^ and those fractions where DNA was confirmed were subjected to fingerprint analysis through DGGE.

### Tag encoded 454 Junior amplicon Pyrosequencing

For pyrosequencing 16S rRNA gene fragments were amplified through nested PCR using primer pair 27f (5′-GAGTTTGATCMTGGCTCAG-3′) and 1492r (5′-AAGGAGGTGATCCANCCRCA-3′). The PCR reaction mixture contained 1 µM of forward and reverse primers, 0.2 mM dNTP’s, 1x reaction buffer (Thermo Scientific), 2.5 u *pfu* polymerase (Thermo Scientific) and 1 µL of template DNA. Reaction conditions were as follows; 95 °C for 5 mins followed by 25–27 cycles of 95 °C for 30 s, 56 °C for 30 s and 72 °C for 1 min 30 s. Amplicons were analyzed by gel electrophoresis (Biorad) and used as a template for nested PCR using primers 909f (5′-ACTCAAAKGAATWGACGG-3′) and 1429r (5′-NTACCTTGTTACGACT-3′), described previously by Berry *et al*.^54^ with an 8 bp (Fusion) identification tag relating to each sample in this study (Supplementary Table 1), 0.2 mM dNTP’s, 1x reaction buffer and 2.5 u *pfu* polymerase (Thermo Scientific). Nested reaction conditions were as follows; 95 °C for 5 min followed by 5 cycles of 95 °C for 30 s, 52 °C for 45 s and 72 °C for 1 min 30 s. A final extension step of 72 °C was performed for 7 min. Tagged amplicons were analysed by gel electrophoresis and gel purified (Roche) prior to quantification and equimolar 454 sequencing.

### Barcoded pyrosequencing analysis

Amplicons for pyrosequencing were submitted to the sequencing facility in the Department of Biochemistry, Cambridge University where pyrosequencing was performed using a Roche 454 Junior sequencer. The 16S rDNA amplicons were generated with primer pair 909F (SEQ) and 1492R (SEQ)^28^. Primer X was synthesized with unique tags to allow for sample identification^55^. Sequences were analysed using the Quantitative Insights Into Microbial Ecology (QIIME) as described by Caporaso *et al*.^56^. Following quality checking for the removal of chimeras and ambiguous bases a total of 22474 reads were attributed to the samples used in this study which were clustered into relevant OTU groups according to sequence similarity-predetermined at a level of 97%^57^. Representative OTU sequences were picked, and aligned using the PyNast algorithm^56^ prior to taxonomy assignment using the GreenGenes and RDP classifier^58^. The statistical validity of the pyrosequencing was checked through alpha diversity analysis. All pyrosequencing yielded at least 2000 sequences per sample, and analysis of Shannon diversity suggested that at 400 sequences there was sufficient analysis to suggest representative populations in the 16S rRNA gene populations (data not shown).

### Extraction and analysis of Phospholipid fatty acids (PLFAs)

PLFAs were analysed prior to incubation (T0), at four weeks (T4), eight weeks (T8) and twelve weeks (T12) at the end of the incubation. For extraction of PLFAs, analysis with GCMS-IRMS and statistical analysis please see Hart *et al*., 2013a^12^. T-tests were also used to confirm the statistical significance of changes in the mean δ^13^C values of soil PLFAs over time, using PAST statistical software^59^.

### Soil chemical analysis

#### Elemental analysis

A CHN combustion analyser (Exeter Analytical CE440 elemental analyser) was used to determine the elemental composition (performed in triplicate). For carbon, samples were treated with 1N HCl in Ag capsules following the procedure of Verardo *et al*. (1990) to remove carbonate^60^. After drying overnight, the capsules were wrapped in Sn boats and combusted in the presence of O_2_. The CO_2_ evolved was measured and the TOC content (%) calculated by comparison with a certified reference standard (acetanilide), which was analysed in conjunction with the samples. Phosphorus (P) was analysed by wet digestion according to April and Kokoasse (2009)^61^.

#### Sulfate

Sulfate was measured in triplicate according to established BaCl_2_ turbidimetric methods for soil^62^. Briefly, 1 g (accurately weighed) of soil was extracted with 1% NaCl solution on a horizontal shaker for 2 h. Approximately 60 mg activated charcoal was added and the mixture was filtered. 1 mL of the extract was diluted in 9 ml ultra pure H_2_O. Excess BaCl_2_ powder was added, followed by 2–3 drops of glycerol, and the mixture was stirred for exactly 1 min. The absorbance at 420 nm was measured on a Shimadzu UV1800 UV/Vis spectrophotometer after 2 and 3 min. The maximum absorbance was taken. Sulfate concentration was calculated by regression (R^2^ = 0.997) using a range of sulfate standards from 0 to 75 ppm. The method recovery was assessed using a positive control of Na_2_SO_4_ dissolved in 1% NaCl (60 ppm sulfate ion), and ultra pure H2O (negative control) was used as a method blank.

#### Sulfur

Sulfur and other soil micronutrients such as zinc (Zn), copper (Cu), iron (Fe) and manganese (Mn) were measured by X-Ray Fluorescence (XRF). XRF was performed using a non-destructive portable XRF analyser. The performance of this instrument for soil has previously been assessed^63, 64^. Soil was oven-dried at 50 °C for 48 h, ground by mortar and pestle and passed through a 0.85 mm mesh sieve. Prior to sample analysis, an internal instrument calibration was performed. All samples were analysed in sample holders using the bulk mode for soil sample according to manufacturer specifications. Each sample was analysed for 60 s per sample and in triplicate.

#### pH

The pH of soil samples was obtained by using a standard 1:5 dilution of soil:water^65^.

### Data Availability

The datasets generated during and/or analysed during the current study are available from the corresponding author on reasonable request.

## Electronic supplementary material

Supplementary Information
