# Supplementary material for: Large perturbations in CO2 flux and subsequent chemosynthesis are induced in agricultural soil by the addition of elemental sulfur
Source: Sci Rep. 2017 Jul 5;7:4732. doi: 10.1038/s41598-017-04934-9 (PMC5498539; doi:10.1038/s41598-017-04934-9)
Supplement: Supplementary file 1 — Supplementary Information [file 41598_2017_4934_MOESM1_ESM.pdf]

SUPPLEMENTARY INFORMATION.

Title: Large perturbations in CO_2 flux and subsequent chemosynthesis are induced in agricultural soil by the addition of elemental sulfur.

Authors:
Brian P. Kelleher*†, Paul V. Flanagan‡, Kris M. Hart†, Andre J. Simpson§, Seth F. Oppenheimer ‖, Brian T. Murphy†, Shane S O'Reilly†, Sean F. Jordan†, Anthony Grey†, Aliyu Ibrahim‡ and Christopher C. R. Allen ‡*.

Affiliations:

†School of Chemical Sciences, Dublin City University, Glasnevin, Dublin 9, Ireland
‡The School of Biological Sciences, Queen's University Belfast, Medical Biology Centre, Lisburn Road, Belfast, BT9 5AG, Northern
Ireland
§Department of Chemistry, Division of Physical and Environmental Science, University of Toronto at Scarborough, 1265 Military
Trail, Toronto, Ontario M1C 1A4, Canada
‖Department of Mathematics and Statistics, Shackouls Honors College, Mississippi State University, Mississippi State, Mississippi
39762, United States
* Corresponding authors.

Corresponding author email addresses:
1. Brian Kelleher (brian.kelleher@dcu.ie)
2. Chris Allen (c.allen@qub.ac.uk)

Supplementary Figures:

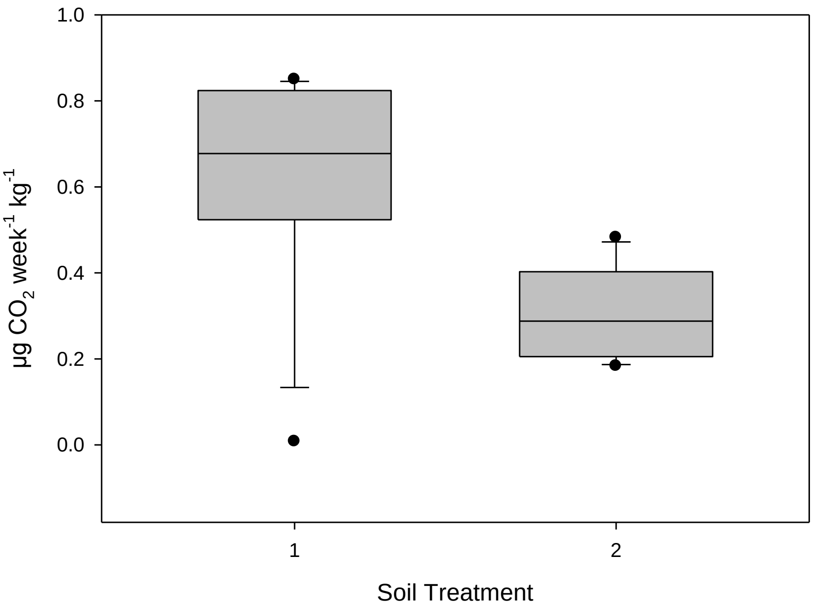

Figure S1. Box and Whisker plot demonstrating the variability of the data. This illustrates that the median values are quite different with soil treatment 1 having much greater variability. Soil Treatment 1 = Soil and Sulfur (S^{SA}), Soil Treatment 2 = Control Soil (S^{U}).

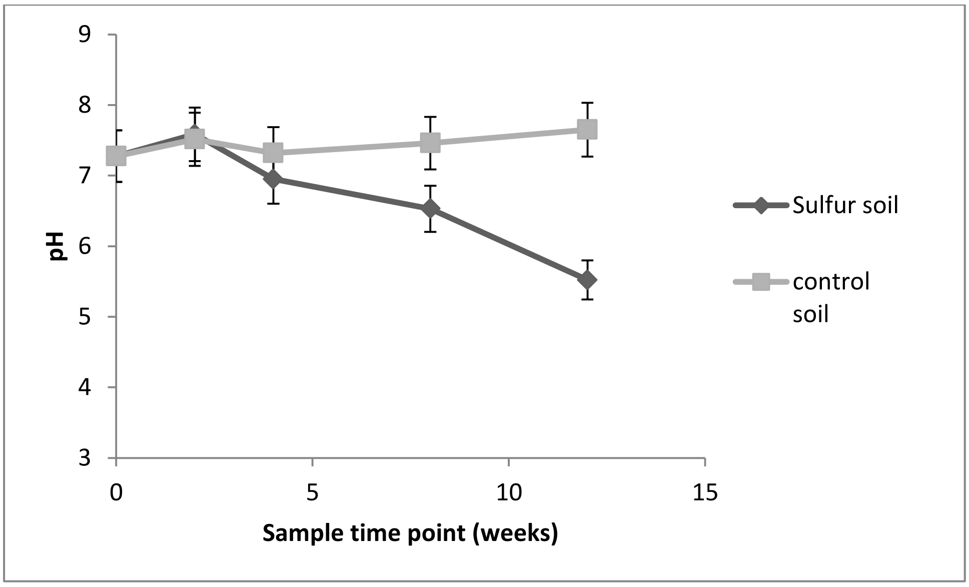

Figure S2. Changes in pH for both the sulfur amended (S^{SA}) and control soils (S^{U}) over the 12 week incubation.

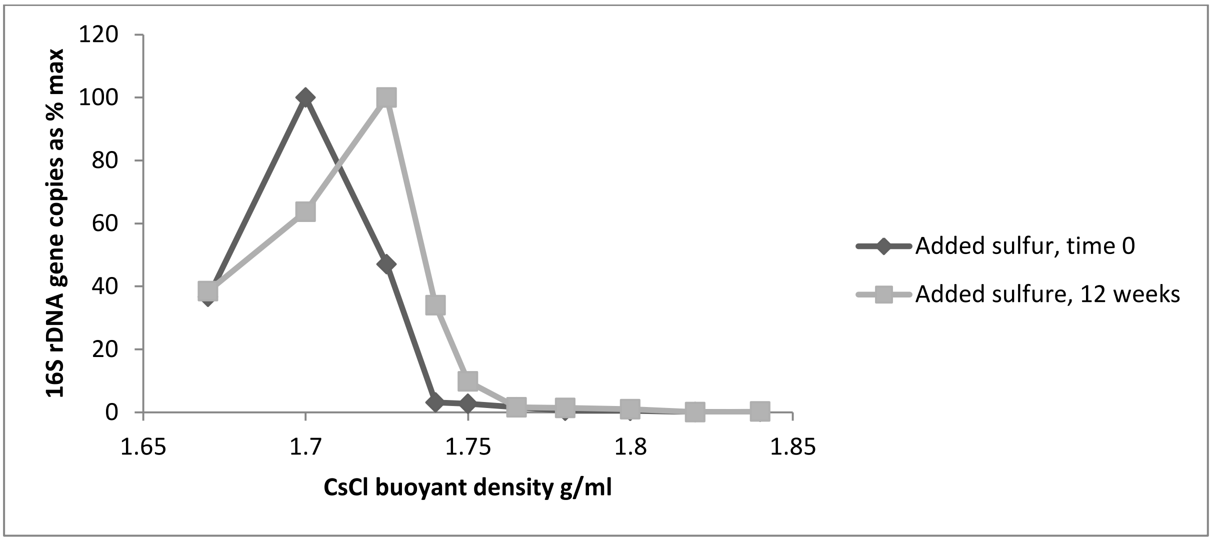

Figure S3. Shift in 16S rDNA gene profile as a result of ^{13}C labelling of DNA

Supplementary Tables

Sample identity	Barcode tag
PF1.TT0	AACCAACC
PF2.TT4A	AACGATCC
PF3.TT4B	AAGCATCC
PF4.TT8A	CCTAATCC
PF5.TT8B	ATCCTACC
PF.TT12A	CGTTATCC
PF.TT12B	TACCATCC

Table S1: ID tags used to identify the pyrosequencing data from each sample. Samples are aligned with their associated barcode tags allowing for multiplexed sequencing to be performed.

† Soil amended with sulfur is identified by the letter A
‡ Soil unaltered is identified with the letter B

Week	Experiment S^{SA} (Sulfur + Soil)	Experiment S^{U} (Soil Only)
#	Average rate of change (ng min^{-1})	Average rate of change (ng min^{-1})
1	8.21	22.06
2	8.73	17.85
3	11.00	16.01
4	11.18	14.91
5	10.40	7.59
6	10.52	11.54
7	9.10	7.45
8	10.95	11.65
9	6.72	8.46
10	0.10	6.79
11	7.40	10.66
12	5.63	7.93

Table S2: Rate of change for corrected soil CO_2. Raw data was collected using continuous measurements (every 30 s) of atmospheric pCO_2 flux above the soil surface. Rates are calculated as a direct computation, not an average of data points ((net change in CO_2 at final

time - net change in CO_2 at initial time)/elapsed time). Positive values describe the net efflux of CO_2 from a soil for each week.

Treatment	Mean (µg CO_2 week^{-1} kg^{-1})	Standard Deviations	Confidence Intervals
Soil and Sulfur (S^{SA})	0.63	0.241	(0.52, 0.75)
Soil (S^{U})	0.32	0.131	(0.21, 0.44)

Table S3: Mean CO_2 efflux rates per treatment with confidence intervals (n = 12).

Table S4: Major PLFA concentrations (µg/g) and $\delta^{13}C$ values in S^{SA} (sulfur amended) and S^{U} (unamended control) before incubation (T0) and at weeks 4, 8 and 12.

PLFAs	**Concentration (µg/g soil)**							**$\delta^{13}C$ values (‰)**						
		S^{SA}			S^{U}				S^{SA}			S^{U}		
	T0	T4	T8	T12	T4	T8	T12	T0	T4	T8	T12	T4	T8	T12
*i*14:O	0.236	0.279	0.267	0.201	0.251	0.236	0.190	-34.79	-16.60	-30.11	-24.91	-33.01	-33.35	-33.19
*i*15:O	0.784	1.005	0.941	0.735	0.928	0.815	0.729	-32.76	-7.25	-9.51	-0.74	-18.12	-22.82	-18.17
*a*15:O	0.619	0.776	0.721	0.585	0.730	0.622	0.580	-31.80	-11.32	-18.62	-3.40	-24.61	-25.47	-23.27
*i*16:O	0.318	0.351	0.337	0.274	0.322	0.296	0.255	-32.43	-13.56	-19.04	-14.37	-25.89	-26.77	-23.68
16:1ω9	0.201	0.225	0.229	0.189	0.241	0.198	0.195	-22.99	-2.54	-3.92	-11.33	-6.24	-4.06	0.55
16:1ω7	0.968	1.301	1.345	1.233	0.987	0.773	0.846	-34.54	98.26	181.27	285.99	-18.22	-20.46	-8.89
16:1ω5	0.541	0.617	0.560	0.499	0.578	0.491	0.485	-31.81	-0.24	4.81	21.71	-12.23	-10.91	-4.90
16:O	1.276	1.388	1.402	1.223	1.126	1.039	1.017	-32.29	45.59	93.08	184.49	-6.93	-13.10	-5.80
*i*17:1ω7	0.266	0.377	0.361	0.268	0.396	0.310	0.226	-15.06	-5.12	-0.41	16.06	-11.46	-11.02	-18.14
*i*17:O	0.244	0.259	0.238	0.203	0.245	0.208	0.198	-32.51	-7.63	-9.09	-2.41	-19.12	-23.26	-18.20
*a*17:O	0.250	0.262	0.236	0.216	0.249	0.216	0.198	-30.13	-12.54	-22.63	-26.51	-24.52	-25.54	-22.98
cy17:O	0.372	0.489	0.511	0.480	0.438	0.355	0.365	-34.37	22.78	140.45	188.50	-30.89	-29.75	-24.15
br17:O	0.252	0.347	0.338	0.256	0.299	0.263	0.244	-39.66	-14.45	-21.48	-13.68	-33.40	-33.01	-31.19
18:2ω6,9	0.348	0.130	0.155	0.107	0.106	0.085	0.075	-25.42	-11.28	2.67	<LOD	-19.71	-19.04	<LOD
18:1ω9	0.954	0.956	0.901	0.795	0.852	0.738	0.760	-29.81	-10.63	-16.23	-14.33	-23.60	-22.51	-22.72
18:1ω7	1.613	1.598	1.503	1.507	1.341	1.273	1.328	-36.29	11.87	30.54	44.74	-11.79	-13.39	-5.96
18:1ω5	0.194	0.148	0.135	0.125	0.133	0.130	0.116	-33.92	-12.57	-20.96	-17.12	-28.67	-28.26	-25.39
18:O	0.644	0.402	0.363	0.337	0.359	0.387	0.332	-30.79	-5.36	-6.43	3.55	-18.27	-21.85	-16.45
br18:O	0.287	0.370	0.356	0.287	0.319	0.314	0.254	-31.30	-14.81	-23.29	-20.21	-28.68	-27.54	-25.91
cy19:O	0.644	0.679	0.623	0.557	0.630	0.600	0.575	-35.78	-18.16	-31.55	-29.25	-34.45	-33.09	-31.55
Average	0.551	0.598	0.576	0.504	0.526	0.467	0.448	-31.74	1.35	11.42	29.83	-21.58	-22.43	-18.95

1. CO_2 efflux.

The net change in atmospheric CO_2 produced by the incubated soil over a period of twelve weeks was continuously measured. All external influences of CO_2 were removed after careful measurements of background artefacts. The remaining data represented the net change in CO_2 within the internal chamber atmosphere, due solely to soil gas flux activity. Treatments S^U and S^{SA} were measured to a high degree of resolution (ng min^{-1}) to determine the average rate of change (See Supplementary Information Table 2) and hence the efflux rates for each week (Fig. 1A). Average efflux rates per week (n = 12) of CO_2 for the sulfur amended soil (S^{SA}) and control (S^U) were, 0.63 ± 0.24 and 0.32 ±0.09 µg CO_2 $week^{-1}$ kg^{-1}, respectively (Fig. 1A, Supplementary Information Table 3). The total mass of CO_2 produced by both treatments were 7.59 and 3.86 µg CO_2 kg^{-1}, respectively.

S^{SA} produced an initial rapid increase in CO_2 efflux, almost double that of S^U, up to week nine (Fig. 1A). The average CO_2 efflux rate then fell after week nine, with the period of lowest CO_2 efflux at week ten (0.01 µg CO_2 $week^{-1}$ kg^{-1}), which was concurrent with other data presented here. The rate of efflux for S^U represented normal background respiration under constant temperature and water holding capacity. For the first four weeks of incubation the rate of CO_2 efflux was on average 0.48 ± 0.08 µg CO_2 $week^{-1}$ kg^{-1}. For the remaining eight weeks the rate of CO_2 efflux settled down to a mean value of 0.24 ± 0.05 µg CO_2 $week^{-1}$ kg^{-1}. Therefore, after homogenisation and wetting, S^U appeared to establish a relatively steady state of efflux after ~four weeks incubation.

At week one, both treatments had very similar efflux rates (4.5% difference), indicative of their shared origin from a single homogenised soil sample. The rates of efflux between treatments diverged after week one by 50.8%, most likely due to the stimulatory effect of added S^0. The box and whisker plot (Fig.S1) illustrates the difference between the two treatment medians and clearly shows the variability induced by sulfur addition. Outlier for the sulfur amended soil ($S^{SA)}$ was identified as week ten, demonstrating the extreme reduction of efflux rates during that time period. Overall, the two treatments were significantly different over the course of the experiment ($p = < 0.001$) and we estimate that sulfur addition produced the lowest rate of CO_2 efflux at week ten before returning to background levels of respiration.

2. $^{13}CO_2$ tracking with compound specific stable isotope ratio mass spectrometry (CSIA)

Combining ^{13}C isotope studies with compound specific stable isotope ratio mass spectrometry (CSIA) of phospholipid fatty acids (PLFA) provides taxonomic and quantitative information on the microorganisms utilising a given ^{13}C labelled substrate[1,2,3]. PLFA biomarkers are found solely in the cell membrane of living organisms. On cell death the PLFA's degrade rapidly; therefore PLFA's are biomarkers for viable cells only (White et al. 1979)[4]. The specificity of PLFA's as biomarkers for active microbes is improved by stable isotope studies as microbes utilising the ^{13}C labelled substrate incorporate ^{13}C into their PLFA's (Evershed et al. 2006)[5]. PLFA's are identified by gas chromatography mass spectrometry (GCMS) and their $\delta^{13}C$ values are measured by compound specific gas chromatography- combustion -isotope ratio mass spectrometry (GC-C-IRMS). Here we measured $\delta^{13}C$ values of PLFA's from the incubated soils to assess the impact of sulfur addition on soil autotrophy.

The average $\delta^{13}C$ values of all PLFA's in soils S^U and S^{SA} at the different time points are plotted in Figure 1 (Table S4). The higher $\delta^{13}C$ values obtained for PLFA's at weeks 4, 8 and 12 confirms incorporation of $^{13}CO_2$ into both the S^U and S^{SA} soils. However, the S^{SA} soil (added sulfur) has significantly higher $\delta^{13}C$ values at T4, T8 and T12 compared to S^U (no added sulfur) indicating higher levels of CO_2 sequestration by soil microbes in S^{SA}. The average $\delta^{13}C$ values of the S^U soil increase from -31.74‰ to -21.58‰ between T0 and T4

(P<0.05) but then appear to stabilise and do not increase or decrease significantly (P>0.05) between T4, T8 or T12. The average $\delta^{13}C$ values of the S^{SA} soil also increase (P<0.001) between T0 and T4. However, in contrast to S^U, S^{SA}'s $\delta^{13}C$ values do not stagnate and $^{13}CO_2$ incorporation continues to increase (P<0.05) from 1.35‰ (T4) to 11.42‰ (T8) and a final value of 29.83‰ (T12). In comparison the S^U soil has a much lower $\delta^{13}C$ value of -22.43‰ at the end point (T12).
The abundances of the PLFA's reported do not change significantly from T0 to T12 in either soil, reflecting the sequencing results (Table 4 supplementary data). Of the PLFA's not reported here, there is a notable decrease in the abundance of polyunsaturated fatty acids 20:4ω6, 20:5ω3 and 20:3ω6, which are often used as biomarkers for species of protozoa[6,7,8]. These PLFA's decrease by a factor of 10 between T0 and T4, indicating that the incubation conditions do not favour these organisms.

Over the 12 week incubation of soil S^{SA} the majority of the ^{13}C label was incorporated into gram negative bacterial PLFA's, 16:1ω7, cy17:0 and 18:1ω7, with a final average $\delta^{13}C$ value of 173.07‰ in the S^{SA} soil compared to -8.69‰ in S^U. 16:1ω7 and 18:ω7 have been reported as key fatty acids in *Pseudomonas* sp. PS+[7]. 18:2ω6 and 18:1ω9 are frequently used as fungal molecular markers[9]. While fungi have incorporated ^{13}C into their fatty acids it is difficult to know if this is due to cross feeding or CO_2 uptake. 16:1ω5 is a signature biomarker for arbuscular mycorrhizal fungi (AMF)[10] and is significantly enriched in the sulfur amended soil (S^{SA}) with a $\delta^{13}C$ value of 21.71‰ at T12. AMF are a major functional group in the sequestration of plant derived C to rhizosphere microorganisms[8] but here they appear to be incorporating ^{13}C into their PLFA's through a different pathway. Iso and anteiso branched C15, C16 and C17 fatty acids are used as biomarkers for gram positive bacteria[11] and incorporation of ^{13}C into their fatty acids was much lower than gram negative and fungal fatty acids, as observed by Butler et al, 2003[3]. The saturated PLFA's 16:0 and 18:0 also show high levels of enrichment, especially in S^{SA} with $\delta^{13}C$ values of 184.49‰ and 3.55‰ respectively. Although these biomarkers are non-specific, 16:0 is an important intermediate in the cellular biosynthesis of fatty acids[3]. Iso17:1ω7 has a final $\delta^{13}C$ value of 16.06‰ and has been reported as a biomarker for sulfate reducing bacteria. Overall, these results show that the addition of sulfur leads to a large increase in ^{13}C incorporation into the PLFA's of gram negative bacteria and fungi.

3. RubisCO gene (*cbbL*) copy abundance.

Over the 12 week incubation RubisCO gene copies (*cbbL*) were monitored (Fig 1E) to establish any variations in abundance over the course of the experiment. The presence of the *cbbL* gene indicates the potential for atmospheric CO_2 uptake through the Benson-Bassham (CBB) reductive pentose phosphate pathway (Tourova et al, 2010)[12]. Prior to incubation, *cbbL* gene copies were determined to be 6.2 X 10^3 $ng^{-1\ soil}$ DNA. Analysis of *cbbL* abundance after 2 weeks shows a negligible rise in S^{SA} to 6.4 X 10^3 ng^{-1} DNA whilst there is a more notable increase in S^U to 7.9 X 10^3 ng^{-1} DNA. The absence of light may encourage microbes that harbour the *cbbl* gene, but the addition of sulfur appears to have temporarily inhibited their growth. At approximately 2.5 weeks however the *cbbL* copies increase rapidly in S^{SA} whilst remaining low in S^U. The beginning of this large increase coincides with the beginning of the transformation of S° to SO_4^{2-} strongly indicating that the SO_4^{2-} is acting as an electron donor. The availability of chemical energy has also stimulated the microbial community which is reflected in an increased production of CO_2 through microbial respiration. The peak in *cbbl* abundance occurs at week 8 (2.3 X 10^4 ng^{-1} DNA), just as the CO_2 uptake event occurs, again strongly suggesting that SO_4^{2-} facilitated chemosynthesis via the CBB pathway is taking place. By week 12 the *cbbL* gene copies decrease sharply in both soils, falling to

levels much lower than those at the beginning of the experiment. Nutrient exhaustion may cause this collapse but CO_2 fixation may still be possible through other pathways.

4. Microbial Community Changes

The microbial phyla identified in both incubated soils resemble those already reported for diverse soils[13]. The most notable changes observed in the experiment involve Acidobacteria and Proteobacteria where increases in the number of sequences representing these phyla were observed over the course of the experiment (Figure 2). Acidobacteria is a complex phylum with few known isolates and is divided into 26 subgroups based on 16S rRNA sequence analysis[14,15]. They were consistently higher in S^U throughout the incubation period with the population peaking at week 8 when 47% of total microbial sequences generated from S^U were attributed to this phylum. Within the phylum, 18 orders were observed over the course of the experiment in both S^U and S^{SA} with order iii1-15 and RB 41, group 5 and group 3 subdivisions respectively, dominating. The relative abundance of Acidobacteria is strongly correlated with pH, particularly within a number of sub groups including group 3 that has a negative relationship[14]. While the abundance of Acidobacteria increased in both soils (S^U S^{SA}), the highest increase occurred in S^U where the pH was stable at 7. These micro-organisms are known to have slow metabolic rates that allow them to survive in low nutrient environments[16]. The unamended soil (S^U) had no new source of nutrients over the 12 week experiment and therefore is relatively nutrient depleted compared to S^{SA} soil.

Thiobacillus spp flourished in S^{SA} but not in S^U where they remain below detection limits from week 8 onwards. The addition of sulfur has stimulated an expansion of the *Thiobacillus spp*. The Genera *Thiobacillus* is a diverse one with species capable of growth at low pH[17], under aerobic and anaerobic conditions[18,19], with the capacity to grow heterotrophically[20,21], chemoautrophically[22],[23] and the capability of oxidising elemental sulfur[24,25] and iron [26], they are therefore also considered as mixotrophs. The *Thiobacillus* genera are flexible and their elevated presence within S^{SA} suggests that they are oxidizing S^0 and/or growing via a chemoautotrophic lifestyle.

Less dominant phyla present in both S^U and S^{SA} throughout the incubation period include candidate division WS3, recently designated Latescibacteria[27], Nitrospirae and Planctomycetes detected at low levels, varying from 0.5% - 5% of the total microbial population. The phylum *Latescibacteria* has no isolated representatives[28] but sequences sharing homology with this phylum have been detected in varying environments including marine sediments obtained at depths >400 m[29], surface sediments of fresh water lakes[30] and hydrocarbon contaminated aquifers[31]. One representative of the latescibacteria phylum has recently been shown to contain the RubisCO gene[27]. The Phylum *Nitrospirae* is also made up of genera with the capacity to grow under chemoautotrophic conditions[32].

5. DNA labelling provides insights into carbon fixing populations

The species identified within the most abundant genus (*Thiobacillus spp)* were: *Thiobacillus thioparus, Thiobacillus denitrificans, Thiobacillus thiophillus* and *Thiobacillus k6.* Thiobacillus species have been identified and isolated from agricultural soils in diverse locations across the world[33], and their importance is based on the fact that S° must be oxidised to SO_4^{-2} to aid plant growth. *Thiobacillus denitrificans* is interesting as not only is it one of the first bacteria to be linked to sulfur-based chemoautotrophy[34], but unlike others of the *Thiobacillus* genera who perform aerobic respiration, T. denitrificans can use nitrate as the electron acceptor under anaerobic conditions if necessary[35]. The remaining 37.3% of isotopically enriched sequences could not be identified beyond the family level suggesting

the presence of sequences that may be divergent in comparison to current database representatives.

References

1. Amelung, W., Bol, R. & Friedrich, C. Natural 13C abundance: a tool to trace the incorporation of dung-derived carbon into soil particle-size fractions. *Rapid Commun. Mass Spectrom. RCM* **13,** 1291–1294 (1999).
2. Butler, J. L., Williams, M. A., Bottomley, P. J. & Myrold, D. D. Microbial Community Dynamics Associated with Rhizosphere Carbon Flow. *Appl. Environ. Microbiol.* **69,** 6793–6800 (2003).
3. Treonis, A. M. *et al.* Identification of groups of metabolically-active rhizosphere microorganisms by stable isotope probing of PLFAs. *Soil Biol. Biochem.* **36,** 533–537 (2004).
4. White D.C. Davis W. M., Nickels, J. S., King, J. D., and Bobbie, R. J. Determination of the sedimentary microbial biomass by extractable lipid phosphate. *Oecologia* **40,** 51–62
5. ^{13}C-Labelling of lipids to investigate microbial communities in the environment. Available at: http://www.sciencedirect.com/science/article/pii/S095816690600005X. (Accessed: 12th August 2016)
6. Cavigelli, M. A., Robertson, G. P. & Klug, M. J. Fatty acid methyl ester (FAME) profiles as measures of soil microbial community structure. *Plant Soil* **170,** 99–113 (1995).
7. Mauclaire, L., Pelz, O., Thullner, M., Abraham, W.-R. & Zeyer, J. Assimilation of toluene carbon along a bacteria-protist food chain determined by 13C-enrichment of biomarker fatty acids. *J. Microbiol. Methods* **55,** 635–649 (2003).
8. Drigo, B. *et al.* Shifting carbon flow from roots into associated microbial communities in response to elevated atmospheric CO2. *Proc. Natl. Acad. Sci.* **107,** 10938–10942 (2010).

9. Frostegård, A. & Bååth, E. The use of phospholipid fatty acid analysis to estimate bacterial and fungal biomass in soil. *Biol. Fertil. Soils* **22,** 59–65

10. Olsson, P. A., Aarle, I. M. van, Gavito, M. E., Bengtson, P. & Bengtsson, G. 13C Incorporation into Signature Fatty Acids as an Assay for Carbon Allocation in Arbuscular Mycorrhiza. *Appl. Environ. Microbiol.* **71,** 2592–2599 (2005).

11. Zelles, L. Fatty acid patterns of phospholipids and lipopolysaccharides in the characterisation of microbial communities in soil: a review. *Biol. Fertil. Soils* **29,** 111–129

12. Tourova, T. P., Kovaleva, O. L., Sorokin, D. Y. & Muyzer, G. Ribulose-1,5-bisphosphate carboxylase/oxygenase genes as a functional marker for chemolithoautotrophic halophilic sulfur-oxidizing bacteria in hypersaline habitats. *Microbiol. Read. Engl.* **156,** 2016–2025 (2010).

13. Roesch, L. F. W. *et al.* Pyrosequencing enumerates and contrasts soil microbial diversity. *ISME J.* **1,** 283–290 (2007).

14. Jones, R. T. *et al.* A comprehensive survey of soil acidobacterial diversity using pyrosequencing and clone library analyses. *ISME J.* **3,** 442–453 (2009).

15. Quaiser, A. *et al.* Acidobacteria form a coherent but highly diverse group within the bacterial domain: evidence from environmental genomics. *Mol. Microbiol.* **50,** 563–575 (2003).

16. Ward, N. L. *et al.* Three genomes from the phylum Acidobacteria provide insight into the lifestyles of these microorganisms in soils. *Appl. Environ. Microbiol.* **75,** 2046–2056 (2009).

17. Rawlings, D. E. & Kusano, T. Molecular genetics of Thiobacillus ferrooxidans. *Microbiol. Rev.* **58,** 39–55 (1994).

18. Subletta, K. L. Aerobic oxidation of hydrogen sulfide by Thiobacillus denitrificans. *Biotechnol. Bioeng.* **29,** 690–695 (1987).

19. Pronk, J. T., Meesters, P. J. W., Dijken, J. P. van, Bos, P. & Kuenen, J. G. Heterotrophic growth of Thiobacillus acidophilus in batch and chemostat cultures. *Arch. Microbiol.* **153,** 392–398

20. Wood, A. P. & Kelly, D. P. Heterotrophic growth of Thiobacillus A2 on sugars and organic acids. *Arch. Microbiol.* **113,** 257–264 (1977).

21. Pronk, J. t., Meulenberg, R., Hazeu, W., Bos, P. & Kuenen, J. g. Oxidation of reduced inorganic sulphur compounds by acidophilic thiobacilli. *FEMS Microbiol. Lett.* **75,** 293–306 (1990).

22. Léjohn, H. B., Van Caeseele, L. & Lees, H. Catabolite repression in the facultative chemoautotroph Thiobacillus novellus. *J. Bacteriol.* **94,** 1484–1491 (1967).

23. Vlasceanu, L., Popa, R. & Kinkle, B. K. Characterization of Thiobacillus thioparus LV43 and its distribution in a chemoautotrophically based groundwater ecosystem. *Appl. Environ. Microbiol.* **63,** 3123–3127 (1997).

24. Suzuki, I., Chan, C. W. & Takeuchi, T. L. Oxidation of Elemental Sulfur to Sulfite by Thiobacillus thiooxidans Cells. *Appl. Environ. Microbiol.* **58,** 3767–3769 (1992).

25. YANG, Z.-H., STÖVEN, K., HANEKLAUS, S., SINGH, B. R. & SCHNUG, E. Elemental Sulfur Oxidation by Thiobacillus spp. and Aerobic Heterotrophic Sulfur-Oxidizing Bacteria. *Pedosphere* **20,** 71–79 (2010).

26. Okereke, A. & Stevens, S. E. Kinetics of Iron Oxidation by Thiobacillus ferrooxidans. *Appl. Environ. Microbiol.* **57,** 1052–1056 (1991).

27. Rinke, C. *et al.* Insights into the phylogeny and coding potential of microbial dark matter. *Nature* **499,** 431–437 (2013).

28. Ikenaga, M., Guevara, R., Dean, A. L., Pisani, C. & Boyer, J. N. Changes in community structure of sediment bacteria along the Florida coastal everglades marsh-mangrove-seagrass salinity gradient. *Microb. Ecol.* **59,** 284–295 (2010).

29. Zeng, Y. *et al.* Phylogenetic diversity of sediment bacteria in the northern Bering Sea. *Polar Biol.* **34,** 907–919 (2011).

30. Tamaki, H. *et al.* Comparative Analysis of Bacterial Diversity in Freshwater Sediment of a Shallow Eutrophic Lake by Molecular and Improved Cultivation-Based Techniques. *Appl. Environ. Microbiol.* **71,** 2162–2169 (2005).

31. Dojka, M. A., Hugenholtz, P., Haack, S. K. & Pace, N. R. Microbial Diversity in a Hydrocarbon- and Chlorinated-Solvent-Contaminated Aquifer Undergoing Intrinsic Bioremediation. *Appl. Environ. Microbiol.* **64,** 3869–3877 (1998).

32. *Bergey's Manual® of Systematic Bacteriology*. (Springer New York, 2001).

33. Chapman, S. J. Thiobacillus populations in some agricultural soils. *Soil Biol. Biochem.* **22,** 479–482 (1990).

34. Beijebinck, M. W. *Pl6nom6nes de reduction produits par les microbes. Archives des Sciences Exactes et Naturelles. Haarlem*. (1904a).

35. Smith, D. W. & Strohl, W.R. in *Variations in Autotrophic Life.* 121–146 (London Academic Press, 1991).
